# The Risk of Being Obese According to Short Sleep Duration Is Modulated after Menopause in Korean Women

**DOI:** 10.3390/nu9030206

**Published:** 2017-02-27

**Authors:** Miae Doo, Yangha Kim

**Affiliations:** Department of Nutritional Science and Food Management, Ewha Womans University, Ewhayeodae-gil, Seodaemun-gu, Seoul 03760, Korea; miae_doo@ewha.ac.kr

**Keywords:** menopausal status, sleep duration, carbohydrate-rich foods, obesity, Korean national health and nutrition examination survey

## Abstract

We previously reported that women with short sleep duration consumed more dietary carbohydrate and showed an increased risk for obesity compared to those who slept adequately, but not for men. Using a cross-sectional study of 17,841 Korean women, we investigated the influence of sleep duration on obesity-related variables and consumption of dietary carbohydrate-rich foods in relation to menopausal status. Premenopausal women with short sleep duration had significantly greater body weight (*p* = 0.007), body mass index (*p* = 0.003), systolic and diastolic blood pressures (*p* = 0.028 and *p* = 0.024, respectively), prevalence of obesity (*p* < 0.016), and consumption of more carbohydrate-rich foods such as staple foods (*p* = 0.026) and simple sugar-rich foods (*p* = 0.044) than those with adequate sleep duration after adjustment for covariates. Premenopausal women with short sleep duration were more obese by 1.171 times compared to subjects adequate sleep duration (95% confidence interval = 1.030–1.330). However, obesity-related variables, dietary consumption, and odds of being obese did not differ according to sleep duration for postmenopausal women. The findings suggest that the increased risk for obesity and consumption of dietary carbohydrate-rich foods with short sleep duration appeared to disappear after menopause in Korean women.

## 1. Introduction

Sleep duration is regarded as a risk factor for obesity. It is generally accepted that poor sleep duration is associated with higher obesity-related variables and an increased risk for developing obesity as well as being associated with dietary consumption patterns [1,2,3]. However, poor sleep duration is more prevalent in women, and these associations are not comparable between men and women. Interestingly, our previous study reported these associations only for women, and not for men [3]. Women with poor sleep duration were particularly associated with increased odds of being obese among women who consumed more dietary carbohydrate (CHO) than subjects with adequate sleep duration [3]. 

Unlike men, women are greatly affected by hormone fluctuations during the menstrual cycle, which regulate dietary consumption and metabolic and physiological conditions [4]. Menopause is the cessation of reproductive ability in a woman’s life and is associated with a decrease in hormone production [4,5]. The decrease in estrogen levels is known to be associated with behavioral changes such as stress, nervousness, depression, and poor sleep as well as physiological changes [6]. Postmenopausal women tend to show an increased prevalence for poor sleep compared with premenopausal women [7]. However, some studies have reported that poor sleep is not primarily attributed to decreases in the production of estrogen [8,9]. 

Therefore, we hypothesized that the gender differences in the interaction between short sleep duration and dietary consumption in relation to obesity in our previous study was caused by differences in reproductive hormones. To determine this hypothesis, we conducted a study in which menopausal status was divided into two categories, premenopausal and postmenopausal women, by considering the data of women who participated in the Korean National Health and Nutrition Examination Survey (KNHANES). Further, we investigated the influence of sleep duration on obesity-related variables and consumption of dietary CHO-rich foods in relation to menopausal status.

## 2. Subjects and Methods 

### 2.1. Study Population and Subject Selection

**Figure 1:**
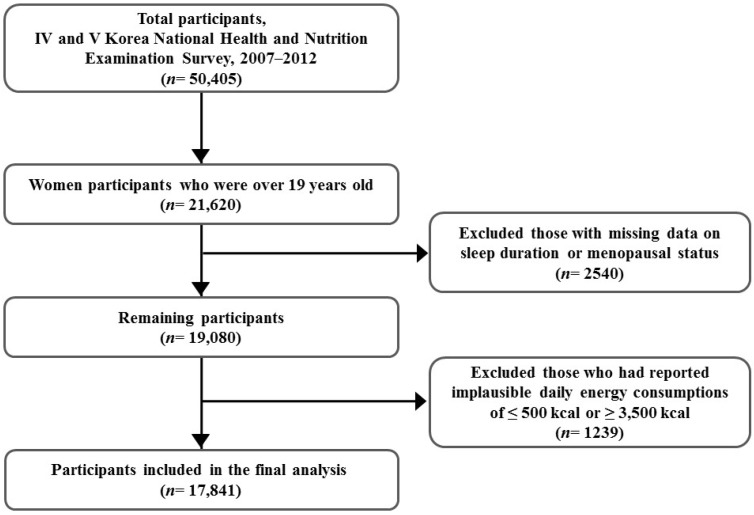
Framework of participants’ selection.

The study was based on data from the KNHANES VI (2007–2009) and V (2010–2012), which is a national representative and cross-sectional survey carried out by the Korea Centers for Disease Control and Prevention (KCDC). The KNHANES was conducted to investigate the health and nutritional status of selective participants from a non-institutionalized civilian Korean population. The survey used a complex and stratified multi-stage probability clustered sampling design and consisted of a health interview, a physical health examination, and a nutritional survey. Detailed information can be found elsewhere [10]. From a total of 50,405 participants (22,926 men and 27,479 women) in the KNHANES IV–V (2007–2012), 21,620 women over the age of 19 years were selected for this study. Women with missing and inadequate sleep duration (*n* = 1690) and menopausal status (*n* = 850) data were excluded. Additionally, women who reported an implausible daily total energy consumption of ≤500 kcal or ≥3500 kcal (*n* = 1239) were excluded. Finally, 17,841 women were selected for the final analysis (Figure 1). Analysis before and after excluding missing and inadequate data showed no significant differences in sleep duration and energy consumption compared with menopausal status. All participants signed the provided written informed consent, and the survey protocol was approved by the Institutional Review Board of the KCDC.

### 2.2. Measurements

Menopausal status, anthropometric and blood biochemical factors, sleep duration, and dietary consumption were collected from the KNHANES data. Menopausal status and sleep duration data from the health interview and dietary consumption data from the nutritional survey were collected by trained specialists using questionnaires. Anthropometric and blood biochemical variables were obtained from the health examination using a direct measurement method.

### 2.3. Menopausal Status

Menopausal status was determined based on the following question: “Have you menstruated for the past 12 consecutive months?” If “yes”, participants were defined as premenopausal, and if “no”, participants were defined as postmenopausal.

### 2.4. Anthropometric Variables and Obesity Definition

Anthropometric variables were measured directly as part of the health examination [11]. Height and body weight were measured with participants wearing light clothing and no shoes to the nearest 0.1 cm using a stadiometer (SECA 225; seca GmbH, Co., KG., Hamburg, Germany) and 0.1 kg using an electronic scale (GL-6000-20; G-tech, Seoul, Korea), respectively. Body mass index (BMI) was calculated by dividing weight (kg) by height squared (m^2^). The definition of obesity (based on criteria from the International Obesity Task Force of adults in the Asia-Pacific region) was BMI > 25 kg/m^2^ [12]. Waist circumference (WC) was measured at the narrowest point between the lower borders of the rib cage and the uppermost borders of the iliac crest at the end stage of a normal expiration to the nearest 0.1 cm using a measuring tape (SECA 200; seca GmbH, Co., KG., Hamburg, Germany).

### 2.5. Biochemical Variables

Blood pressure (BP) was measured using a mercury sphygmomanometer (Baumanometer; WA Baum, Co., Copiague, NY, USA) and an average of three readings with 5-min rest intervals in the sitting position was used for the analysis [11]. Blood samples were collected after an overnight fast. Fasting glucose (FG), total cholesterol (TC), triglycerides (TG), and high-density lipoprotein (HDL-C) were analyzed using a Hitachi 7000 automatic analyzer (Hitachi, Tokyo, Japan) in a certified clinical laboratory [11].

### 2.6. Sleep Duration Assessment

Sleep was assessed using a self-reported questionnaire about average sleeping hours per day. Participants were divided into two categories according to their sleep duration: short sleep duration (≤6.9 h/day) and adequate sleep duration (≥7.0 h/day). A sleep duration of 7–8 h/day was taken as the recommended time for sleeping in accordance with previous studies [13,14].

### 2.7. Dietary Consumption Assessment

Assessment of dietary consumption was conducted by face-to-face interviews by trained dietitians. A single 24-h recall record method was used to obtain food items, which were categorized into 18 food groups based on common food groups classified in the Korean Nutrient Database [15]. Among 18 food groups, the drink group was divided into three subgroups: sweet; non-sugar (e.g., green tea, black tea, pure coffee); and alcohol. Consumption of CHO-rich foods as the staple foods or dessert foods is considered to be generally high in Korean women and to be influenced by sleep duration [3]. As a result, CHO-rich foods were defined as either staple foods such as a combination of grain and potato products or simple sugar-rich foods such as a combination of sugary products and sweet beverages.

### 2.8. Statistical Analyses

To reflect estimates of the entire Korean population, sample weights were applied in all analyses. To evaluate general characteristics and food group consumption by menopausal status, categorical variables, such as obesity prevalence, physical activity, current smoking, and drinking status were analyzed by Pearson’s Chi-square test. Continuous variables, such as age, height, weight, BMI, BP, FG, TG, TC, HDL-C, sleep duration, and dietary consumption were analyzed by independent *t*-tests. Because data were divided by menopausal status, generalized linear models were used to analyze the effects of sleep duration on anthropometric and biochemical variables and on food-group consumption as well as the interaction between menopausal status and sleep duration with respect to the anthropometric and biochemical variables and on food-group consumption after adjustment for covariates. Socioeconomic variables (age, education level, and monthly household income), disease prevalence-related variables (hypertension, cardiovascular disease, and diabetes), and health-related variables (age, smoking status, alcohol drinking status, and physical activity) were adjusted to prevent confounding effects. The interaction between menopausal status and sleep duration on the risk for being obese was determined using a multivariable logistic regression model. According to menopausal status, the odds ratio (OR) and 95% confidence intervals (CI) for the risk for being obese were estimated in reference to sleep duration of ≥7.0 h/day. A *p*-value < 0.05 was considered statistically significant. All statistical analyses were performed using SPSS (version 21.0; IBM Corp., Armonk, NY, USA) software for Windows.

## 3. Results

The general characteristics stratified by menopausal status are shown in Table 1. The average age was 35.71 and 62.85 years for premenopausal and postmenopausal women, respectively, and the percentage of women participating with each menopausal status was 51.9% and 48.1%, respectively. There were significant differences between menopausal statuses for all anthropometric and blood biochemical variables except for weight. BMI, WC, systolic blood pressure (SBP), diastolic blood pressure (DBP), FG, TG, and TC were significantly higher in postmenopausal women, but height and HDL-C were significantly higher in premenopausal women (*p* < 0.001 for all). Postmenopausal women showed higher prevalence of obesity than premenopausal women (*p* < 0.001 for both). Among health-related habits, premenopausal women showed a higher proportion who currently smoked and drank alcohol compared with menopausal women (*p* < 0.001 for both). Sleep duration was significantly higher in premenopausal compared with postmenopausal women (7.06 and 6.47 h, respectively, *p* < 0.001).

Among the different food groups, consumption of grains, potatoes, sugar, nuts, vegetables, fruit, eggs, fish, seaweed, milk, oil, seasoning, sweet drinks, tea, and alcohol were significantly different according to menopausal status (Table 2). The staple foods (177.49 ± 3.61 g vs. 166.43 ± 2.77 g, *p* < 0.001) were consumed more, but simple sugar foods (84.95 ± 4.67 g vs. 79.95 ± 2.92 g, *p* < 0.001) were consumed less in postmenopausal women compared with premenopausal women. 

**Figure 2:**
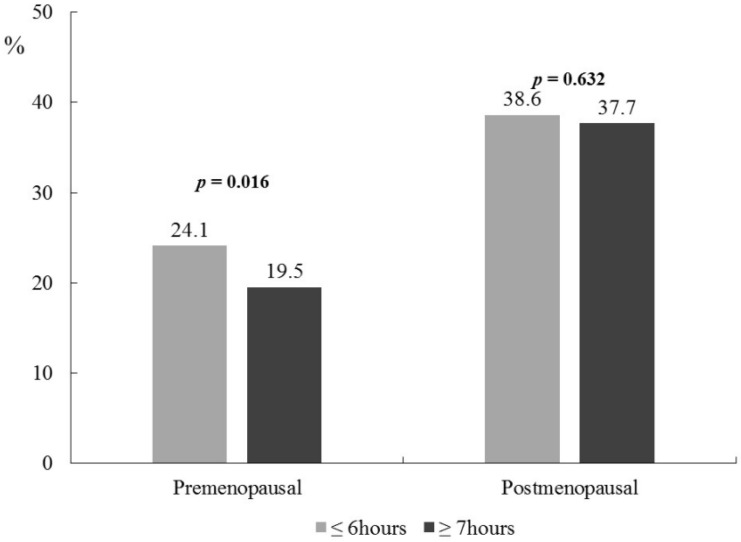
Prevalence of obesity and metabolic syndrome by sleep duration in participating Korean women. Premenopausal, *n* = 2906 for ≤6 h, *n* = 6308 for ≥7 h. Postmenopausal, *n* = 4297 for ≤6 h, *n* = 4276 for ≥7 h. *p*-values were calculated using a general linear model test after adjustment for age, education level, monthly household income, hypertension, cardiovascular disease, diabetes, smoking status, alcohol drinking, and physical activity

Significant differences in obesity-related variables and BP by sleep duration were observed in premenopausal women after adjustment for age, education level, monthly household income, hypertension, cardiovascular disease, diabetes, smoking status, alcohol drinking, and physical activity (Table 3). Premenopausal women with short sleep duration had significantly higher body weight (58.09 ± 0.22 kg vs. 57.04 ± 0.15 kg, *p* = 0.007) and BMI (22.96 ± 0.08 kg/m^2^ vs. 22.39 ± 0.06 kg/m^2^, *p* = 0.003) compared with those with adequate sleep duration after adjustment for the covariates. Moreover, SBP (109.68 ± 0.28 mmHg vs. 107.76 ± 0.21 mmHg, *p* = 0.028) and DBP (72.54 ± 0.21 mmHg vs. 71.26 ± 0.16 mmHg, *p* = 0.024) were higher among premenopausal women with short sleep duration compared with those with adequate sleep duration. All anthropometric and blood biochemical variables by sleep duration were not significantly different for postmenopausal women, except for HDL-C (*p* = 0.005). Significant interactions between menopausal status and sleep duration were observed for body weight, BMI, SBP, DBP, TC, and HDL-C (*p*-interaction < 0.001 for all), WC (*p*-interaction = 0.008), and TG (*p*-interaction = 0.015). The prevalence of obesity according to sleep duration was significantly different only for premenopausal women and not for postmenopausal women; premenopausal women with short sleep duration showed a higher proportion with obesity after adjustment for the covariates (24.1% vs. 19.5%, *p* = 0.016, Figure 2).

Significant differences in food-group consumption by sleep duration were observed only for premenopausal women, but not for postmenopausal women after adjustment for the covariates (Table 4). Among premenopausal women, those with short sleep duration showed significantly higher consumption of milk and milk products (*p* = 0.042), seasoning (*p* = 0.020), sweet beverages (*p* = 0.027), and tea (*p* = 0.013) compared with those with adequate sleep duration. Only among premenopausal women was short sleep duration associated with the consumption of CHO-rich foods in the adjusted model. In other words, premenopausal women with short sleep duration consumed more CHO-rich foods with respect to the staple foods (177.49 ± 3.61 g vs. 166.43 ± 2.77 g, *p* = 0.026) and simple sugar-rich foods (84.95 ± 4.67 g vs. 79.95 ± 2.92 g, *p* = 0.044) compared with those with adequate sleep duration. Interactions between menopausal status and sleep duration with respect to food-group consumption were found to be significant after adjustment for the covariates (*p*-values for the interaction for potatoes and products, vegetables and products, milk and products, seasoning, sweet drinks, and tea were 0.025, 0.031, 0.038, 0.025, <0.001, and 0.006, respectively). Furthermore, significant interactions between menopausal status and sleep duration by CHO-rich food consumption were observed (*p*-interaction for staple foods and simple sugar-rich foods were 0.028 and <0.001, respectively).

**Figure 3:**
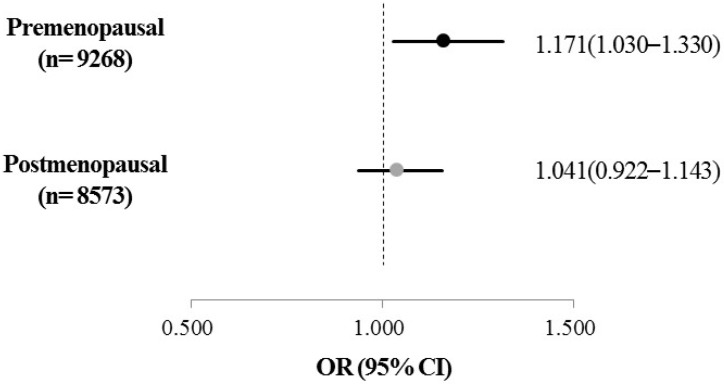
Adjusted odds ratio for obesity and for sleep duration in participating Korean women. OR, odds ratio; CI, confidence interval. The ORs (95% CI) were calculated in reference to sleep duration ≥7.0 h/day using multivariate logistic regression after adjustment for age, education level, monthly household income, hypertension, cardiovascular disease, diabetes, smoking status, alcohol drinking, and physical activity (*P*-interaction between menopausal status and sleep duration = 0.002).

Adjusted OR for the risk for being obese and the interaction between menopausal status and sleep duration were examined using a multivariate logistic regression model after adjustment for age, education level, monthly household income, hypertension, cardiovascular disease, diabetes, smoking status, alcohol drinking, and physical activity (Figure 3). A significant interaction between menopausal status and sleep duration was found to be associated with the risk for being obese (*p*-interaction = 0.002). However, among postmenopausal women, no significant association was observed between obesity and sleep duration (*p* = 0.632). On the other hand, only among premenopausal women with short sleep duration, the adjusted OR for the risk for being obese significantly increased (*p* = 0.016). After adjusting for covariates, premenopausal women with short sleep duration showed a significantly increased risk for being obese (1.171 times) compared with women with adequate sleep duration (95% CI = 1.030–1.330).

## 4. Discussion

In this study, which was based on a national representative Korean women population, obesity-related variables and consumption of CHO-rich foods were found to be different according to menopausal status. Among premenopausal women, sleep duration appeared to influence obesity-related variables, consumption of CHO-rich food, and the risk for being obese in the model adjusted for age, education level, monthly household income, hypertension, cardiovascular disease, diabetes, smoking status, alcohol drinking, and physical activity.

Associations between sleep duration and obesity-related variables in women but not men are reported to be because of hormone differences [3]. In the current study, to investigate hormonal effects, a large population of women was divided by menopausal profile, which was considered to be a critical contributing factor in determining hormonal differences in women. Our finding was consistent with previous studies [16,17,18], which reported an increased trend in the prevalence of obesity after menopause. It is possible that the findings are associated with changes in reproductive hormones, such as estrogen, follicle stimulating hormone (FSH), and luteinizing hormone (LH) as well as melatonin secretion after entering menopause [15,16]. These changes lead to metabolic changes such as increases in appetite, dietary consumption, and adiposity as well as alternation in energy homeostasis [19,20,21]. A study of a population of Brazilian females reported that postmenopausal women with higher BMI levels had high levels of TG and FG but low levels of HDL-C [22].

Generally, for Koreans, the traditional main meal consists of rice and potatoes, and these products, which are consumed two to three times per day as staple food, provide about 37.9% of the total daily energy [23]. In the current study, postmenopausal women consumed more staple food and less simple sugar food compared with premenopausal women. Premenopausal women, who were mostly younger, had a higher consumption of simple sugar food and consumed more grams of food in the form of snacks than as the main meal when compared with postmenopausal women.

Interestingly, after adjustment for age, education level, monthly household income, hypertension, cardiovascular disease, diabetes, smoking status, alcohol drinking, and physical activity, premenopausal women with short sleep duration showed a significant association with both higher obesity-related variables and the risk for being obese, but this was not found for postmenopausal women. Previous clinical studies [24,25,26] have reported that a shorter sleep duration is associated with increases in the intake of dietary CHO or CHO-rich foods, which is consistent with our results in premenopausal women, and may be associated with decreased leptin and increased ghrelin levels [24,25,26]. 

In our previous study, we reported that increased dietary consumption of CHO-rich foods in relation to short sleep duration potentially induced obesity in women, but not in men [3]. The correlation was explained by the combination of appetite controlling hormones such as leptin and ghrelin as well as the reproductive steroid hormones. When circulating estrogen levels decrease, levels of FSH and LH increase [27]. Increases in FSH and LH are reported to be associated with a low blood melatonin level, and injection of melatonin has been shown to reinforce the effects of FSH and LH [28]. Low levels of blood melatonin in the fall–winter period [29], which is a main marker of the circadian system, are reported to increase appetite and influence poor sleep duration [29,30]. Normal levels of estrogen, FSH, and LH in premenopausal women may affect the levels of blood melatonin, which controls adequate sleep duration and status. Moreover, melatonin regulates appetite controlling hormones such as leptin and ghrelin, which are associated with the consumption of CHO-rich foods. It is suggested that the increased risk for obesity caused by short sleep duration in premenopausal women might decrease in postmenopausal women due to abnormal changes in the levels of reproductive hormones, such as estrogen, FSH, and LH.

These interesting data suggest that menopausal status may be related to the consumption of CHO-rich foods, and may therefore influence obesity. However, this study has several limitations. First, the data that is used in this study is from a cross-sectional study. Consequently, the results of this study, per se, could not explain the sequence of associations between variables. Second, the age of the postmenopausal women was substantially higher compared to the premenopausal women. Although age as a confounding factor was adjusted, physical and psychological changes related with age may influence obesity status and dietary intake in addition to menopausal status. Third, we classified two categories for sleep duration; “short” and “proper”. Although some studies [31,32] showed the association of long sleep duration with obesity, the participants with long sleep duration (≥9.0 h/day) were low (8.0%) in this study. Finally, the menopausal status in this study was divided into two categories. However, more detailed menopausal conditions should be considered in future studies. For example, the 3 to 5 years preceding menopause could be taken into consideration because other physiological changes may occur during the menopausal transition period.

## 5. Conclusions 

In conclusion, the results using representative subject data of Korean women from KNHANE, demonstrated that menopausal status is related to the differences in obesity-related variables and consumption of CHO-rich foods. Indeed, short sleep duration was associated with increased levels of obesity-related variables and increased consumption of CHO-rich foods in premenopausal women, but not in postmenopausal women. These findings suggest that the increased risk for being obese and the consumption of dietary CHO-rich foods that are associated with short sleep duration are likely to be modulated after menopause in Korean women.

## Figures and Tables

**Table 1:** General characteristics of Korean women study participants.

	Premenopausal (*n* = 9268)	Postmenopausal (*n* = 8573)	*p*-Value *
Age (years)	35.71 ± 0.14	62.85 ± 0.15	<0.001
Height (cm)	159.46 ± 0.08	153.34 ± 0.09	<0.001
Weight (kg)	57.38 ± 0.13	57.24 ± 0.14	0.460
BMI (kg/m^2^)	22.58 ± 0.05	24.31 ± 0.05	<0.001
WC (cm)	75.37 ± 0.14	82.51 ± 0.17	<0.001
Obesity prevalence (%)	21.0	38.2	<0.001
SBP (mmHg)	108.39 ± 0.18	126.83 ± 0.30	<0.001
DBP (mmHg)	71.68 ± 0.14	77.13 ± 0.15	<0.001
FG (mg/dL)	91.06 ± 0.21	101.50 ± 0.39	<0.001
TG (mg/dL)	96.29 ± 0.99	138.29 ± 1.22	<0.001
TC (mg/dL)	179.34 ± 0.42	201.93 ± 0.55	<0.001
HDL-C (mg/dL)	56.06 ± 0.20	51.43 ± 0.20	<0.001
Sleep duration (h/day)	7.06 ± 0.02	6.47 ± 0.02	<0.001
Physical activity (%)	49.4	47.6	0.050
Current smoking (%)	6.8	4.7	<0.001
Alcohol drinking (%)	49.8	26.1	<0.001

BMI, body mass index; WC, waist circumference; SBP, systolic blood pressure; DBP, diastolic blood pressure; FG, fasting glucose; TG, triglycerides TC, total cholesterol; HDL-C, high density lipoprotein cholesterol. Values are means ± SE or %; * *p*-values calculated using *t*-test or chi-square test.

**Table 2:** Food group consumption in Korean women participants.

	Premenopausal (*n* = 9268)	Postmenopausal (*n* = 8573)	*p*-Value *
Grains and products (g)	134.79 ± 1.69	155.82 ± 2.30	<0.001
Potatoes and products (g)	42.23 ± 1.28	63.69 ± 1.98	<0.001
Sugar and products (g)	4.98 ± 0.12	4.63 ± 0.13	0.034
Tofu and products (g)	30.02 ± 0.74	29.82 ± 0.83	0.844
Nuts and products (g)	2.64 ± 0.13	3.08 ± 0.17	0.030
Vegetables and products (g)	144.29 ± 2.64	159.60 ± 3.23	<0.001
Mushrooms and products (g)	10.65 ± 0.37	10.45 ± 0.57	0.765
Fruits and products (g)	158.49 ± 2.67	169.06 ± 3.16	0.006
Meats and products (g)	54.49 ± 1.05	51.85 ± 1.48	0.122
Eggs and products (g)	22.57 ± 0.44	20.60 ± 0.53	0.003
Fish and shellfishes (g)	29.68 ± 0.59	24.91 ± 0.66	<0.001
Seaweed and products (g)	5.20 ± 0.22	6.81 ± 0.34	<0.001
Milk and products (g)	123.18 ± 2.18	132.71 ± 2.68	0.003
Oil (g)	4.03 ± 0.07	2.83 ± 0.07	<0.001
Seasoning (g)	36.30 ± 2.01	25.21 ± 1.46	<0.001
Sweet beverages (g)	79.48 ± 2.36	45.18 ± 2.05	<0.001
Tea (g)	89.14 ± 4.18	46.01 ± 6.26	<0.001
Alcohol drinks (g)	174.97 ± 6.85	122.77 ± 7.44	<0.001
Staple foods (g)	169.96 ± 2.49	207.29 ± 3.26	<0.001
Simple sugar-rich foods (g)	81.50 ± 2.51	53.20 ± 2.67	<0.001

Values are means ± SE represented as grams per 1000 kcal. * *p*-values calculated using *t*-test.

**Table 3:** Anthropometric and biochemical variables for different sleep duration in participating Korean women.

	Premenopausal (*n* = 9268)				Postmenopausal (*n* = 8573)				*p*-Interaction ^†^
	≤6.9 h (*n* = 2960)	≥7.0 h (*n* = 6308)	*p*-Value *	*p*-Value **	≤6.0 h (*n* = 4297)	≥7.0 h (*n* = 4276)	*p*-Value *	*p*-Value **	
Age (years)	37.28 ± 0.22	34.94 ± 0.16	<0.001	-	63.63 ± 0.21	62.07 ± 0.21	<0.001	-	-
Height (cm)	159.08 ± 0.13	159.64 ± 0.09	<0.001	0.641	153.14 ± 0.12	153.54 ± 0.12	0.019	0.398	0.061
Weight (kg)	58.09 ± 0.22	57.04 ± 0.15	<0.001	0.007	57.20 ± 0.17	57.28 ± 0.20	0.718	0.186	<0.001
BMI (kg/m^2^)	22.96 ± 0.08	22.39 ± 0.06	<0.001	0.003	24.36 ± 0.06	24.27 ± 0.07	0.313	0.374	<0.001
WC (cm)	76.10 ± 0.22	75.02 ± 0.16	<0.001	0.186	82.57 ± 0.20	82.44 ± 0.23	0.633	0.981	0.008
SBP (mmHg)	109.68 ± 0.28	107.76 ± 0.21	<0.001	0.028	126.89 ± 0.37	126.79 ± 0.38	0.844	0.053	<0.001
DBP (mmHg)	72.54 ± 0.21	71.26 ± 0.16	<0.001	0.024	76.99 ± 0.20	77.28 ± 0.19	0.255	0.381	<0.001
FG (mg/dL)	91.67 ± 0.36	90.77 ± 0.24	0.032	0.735	100.90 ± 0.46	102.14 ± 0.58	0.080	0.138	0.059
TG (mg/dL)	96.70 ± 1.37	96.11 ± 1.27	0.746	0.104	136.97 ± 1.70	139.68 ± 1.73	0.260	0.102	0.015
TC (mg/dL)	179.64 ± 0.67	179.21 ± 0.53	0.611	0.071	201.45 ± 0.69	202.42 ± 0.81	0345	0.344	<0.001
HDL-C (mg/dL)	56.13 ± 0.28	56.03 ± 0.23	0.737	0.131	51.73 ± 0.26	51.13 ± 0.26	0.071	0.005	<0.001

BMI, body mass index; WC, waist circumference; SBP, systolic blood pressure; DBP, diastolic blood pressure; FG, fasting glucose; TG, triglycerides TC, total cholesterol; HDL-C, high density lipoprotein cholesterol. Values are means ± SE. * *p*-values calculated using general linear model. ** *p*-values were calculated using general linear model after adjustment for age, education level, monthly household income, hypertension, cardiovascular disease, diabetes, smoking status, alcohol drinking, and physical activity. ^†^ *p* values were obtained in interaction between menopausal statues and sleep duration using general linear model after adjustment for age, education level, monthly household income, hypertension, cardiovascular disease, diabetes, smoking status, alcohol drinking, and physical activity.

**Table 4:** Food group consumption according to sleep duration in participating Korean women.

	Premenopausal (*n* = 9268)				Postmenopausal (*n* = 8573)				*p*-Interaction ^†^
	≤6.9 h (*n* = 2960)	≥7.0 h (*n* = 6308)	*p*-Value *	*p*-Value **	≤6.0 h (*n* = 4297)	≥7.0 h (*n* = 4276)	*p*-Value *	*p*-Value **	
Grains and products (g)	137.50 ± 1.96	133.43 ± 1.88	0.032	0.188	156.39 ± 2.47	155.24 ± 2.68	0.622	0.863	0.198
Potatoes and products (g)	44.67 ± 2.52	41.10 ± 1.42	0.213	0.448	62.45 ± 2.69	65.09 ± 2.85	0.496	0.496	0.025
Sugar and products (g)	5.19 ± 0.22	4.88 ± 0.13	0.199	0.148	4.55 ± 0.16	4.72 ± 0.19	0.472	0.491	0.200
Tofu and products (g)	31.31 ± 1.37	29.38 ± 0.85	0.222	0.317	30.65 ± 1.27	28.97 ± 0.99	0.282	0.478	0.074
Nuts and products (g)	2.75 ± 0.22	2.58 ± 0.16	0.545	0.717	2.93 ± 0.20	3.23 ± 0.27	0.350	0.441	0.405
Vegetables and products (g)	152.09 ± 4.18	140.50 ± 2.77	0.006	0.102	161.16 ± 3.82	157.89 ± 3.82	0.422	0.351	0.031
Mushrooms and products (g)	10.94 ± 0.69	10.51 ± 0.46	0.618	0.670	11.45 ± 0.90	9.52 ± 0.69	0.089	0.143	0.286
Fruits (g)	159.91 ± 4.27	157.83 ± 3.17	0.679	0.794	168.58 ± 4.24	169.54 ± 4.04	0.858	0.848	0.611
Meats and products (g)	55.31 ± 1.67	54.10 ± 1.20	0.518	0.331	52.16 ± 2.11	51.60 ± 1.93	0.839	0.778	0.772
Eggs and products (g)	22.57 ± 0.66	22.57 ± 0.54	0.999	0.719	20.86 ± 0.73	20.35 ± 0.77	0.633	0.677	0.851
Fish and shellfishes (g)	30.30 ± 0.96	29.40 ± 0.69	0.419	0.711	25.18 ± 0.87	24.63 ± 0.87	0.625	0.551	0.152
Seaweed and products (g)	5.21 ± 0.39	5.20 ± 0.24	0.984	0.773	6.48 ± 0.46	7.15 ± 0.45	0.261	0.221	0.612
Milk and products (g)	129.36 ± 3.60	120.36 ± 2.58	0.035	0.042	131.87 ± 3.62	133.45 ± 3.91	0.765	0.725	0.038
Oil and products (g)	3.91 ± 0.10	4.09 ± 0.09	0.113	0.501	2.84 ± 0.08	2.82 ± 0.09	0.853	0.510	0.256
Seasoning (g)	40.44 ± 3.12	34.30 ± 2.08	0.045	0.020	23.92 ± 1.44	26.54 ± 2.26	0.276	0.505	0.025
Sweet beverages (g)	82.23 ± 4.27	78.24 ± 2.67	0.408	0.027	46.19 ± 3.03	44.04 ± 2.76	0.599	0.383	<0.001
Tea (g)	99.58 ± 7.60	83.29 ± 4.74	0.063	0.013	38.68 ± 4.34	53.16 ± 11.55	0.239	0.507	0.006
Alcohol drinks (g)	186.05 ± 11.44	169.12 ± 8.64	0.242	0.559	117.61 ± 8.55	126.66 ± 12.08	0.543	0.287	0.098
Staple foods (g)	177.49 ± 3.61	166.43 ± 2.77	0.004	0.026	205.54 ± 4.17	209.20 ± 4.14	0.477	0.255	0.028
Simple sugar-rich foods (g)	84.95 ± 4.67	79.95 ± 2.92	0.358	0.044	56.23 ± 4.26	50.08 ± 3.13	0.240	0.148	<0.001

Values are means ± SE. * *p*-values were calculated using a general linear model. ** *p*-values were calculated using a general linear model after adjustment for age, education level, monthly household income, hypertension, cardiovascular disease, diabetes, smoking status, alcohol drinking, and physical activity. ^†^ *p* values were obtained in interaction between menopausal statues and sleep duration using a general linear model after adjustment for age, education level, monthly household income, hypertension, cardiovascular disease, diabetes, smoking status, alcohol drinking, and physical activity.

## References

[1] Bayon V., Leger D., Gomez-Merino D., Vecchierini M.F., Chennaoui M. (2014). Sleep debt and obesity. Ann. Med..

[2] Rahe C., Czira M.E., Teismann H., Berger K. (2015). Associations between poor sleep quality and different measures of obesity. Sleep Med..

[3] Doo M., Kim Y. (2016). Association between sleep duration and obesity is modified by dietary macronutrients intake in Korean. Obes. Res. Clin. Pract..

[4] Mihm M., Gangooly S., Muttukrishna S. (2011). The normal menstrual cycle in women. Anim. Reprod. Sci..

[5] Pankaj T. (2014). Manual of Cytogenetics in Reproductive Biology.

[6] Weber M.T., Rubin L.H., Maki P.M. (2013). Cognition in perimenopause: The effect of transition stage. Menopause.

[7] Kravitz H.M., Joffe H. (2011). Sleep during the perimenopause: A SWAN story. Obstet. Gynecol. Clin. N. Am..

[8] Tao M.F., Sun D.M., Shao H.F., Li C.B., Teng Y.C. (2016). Poor sleep in middle-aged women is not associated with menopause per se. Braz. J. Med. Biol. Res..

[9] Young T., Rabago D., Zgierska A., Austin D., Laurel F. (2003). Objective and subjective sleep quality in premenopausal, perimenopausal, and postmenopausal women in the Wisconsin Sleep Cohort Study. Sleep.

[10] Kweon S., Kim Y., Jang M.J., Kim Y., Kim K., Choi S., Chun C., Khang Y.H., Oh K. (2014). Data resource profile: The Korea National Health and Nutrition Examination Survey (KNHANES). Int. J. Epidemiol..

[11] The Fifth Korean National Health and Nutrition Survey (KNHANES V). https://knhanes.cdc.go.kr/knhanes/eng/index.do.

[12] World Health Organization (2000). The Asia- Pacific Perspective: Redefining Obesity and Its Treatment.

[13] Bixler E. (2009). Sleep and society: An epidemiological perspective. Sleep Med..

[14] Gallicchio L., Kalesan B. (2009). Sleep duration and mortality: A systematic review and meta-analysis. J. Sleep Res..

[15] The Korean Nutrition Society (2010). Dietary Reference Intake for Korean.

[16] Colpani V., Oppermann K., Spritzer P.M. (2013). Association between habitual physical activity and lower cardiovascular risk in premenopausal, perimenopausal, and postmenopausal women: A population-based study. Menopause.

[17] Mauvais-Jarvis F., Clegg D.J., Hevener A.L. (2013). The role of estrogens in control of energy balance and glucose homeostasis. Endocr. Rev..

[18] Teede H.J., Lombard C., Deeks A.A. (2010). Obesity, metabolic complications and the menopause: An opportunity for prevention. Climacteric.

[19] Walecka-Kapica E., Klupińska G., Chojnacki J., Tomaszewska-Warda K., Błońska A., Chojnacki C. (2014). The effect of melatonin supplementation on the quality of sleep and weight status in postmenopausal women. Prz Menopauzalny.

[20] Okatani Y., Morioa N., Wakatsuki A. (2000). Changes in nocturnal melatonin secretion in perimenopausal women: Correlation with endogenous estrogen concentrations. J. Pineal Res..

[21] Stachowiak G., Pertyński T., Pertyńska-Marczewska M. (2015). Metabolic disorders in menopause. Prz. Menopauzalny.

[22] Bagnoli V.R., Fonseca A.M., Arie W.M., Das Neves E.M., Azevedo R.S., Sorpreso I.C., Soares Júnior J.M., Baracat E.C. (2014). Metabolic disorder and obesity in 5027 Brazilian postmenopausal women. Gynecol. Endocrinol..

[23] Korea Centers for Disease Control and Prevention (2006). The Report of the Third Korea National Health and Nutrition Examination Survey (KNHANES III) 2005—Nutrition Survey (I).

[24] Markwald R.R., Melanson E.L., Smith M.R., Higgins J., Perreault L., Eckel R.H., Wright K.P. (2013). Impact of insufficient sleep on total daily energy expenditure, food intake, and weight gain. Proc. Natl. Acad. Sci. USA.

[25] Haghighatdoost F., Karimi G., Esmaillzadeh A., Azadbakht L. (2012). Sleep deprivation is associated with lower diet quality indices and higher rate of general and central obesity among young female students in Iran. Nutrition.

[26] Nedeltcheva A.V., Kilkus J.M., Imperial J., Kasza K., Schoeller D.A., Penev P.D. (2009). Sleep curtailment is accompanied by increased intake of calories from snacks. Am. J. Clin. Nutr..

[27] Davis S., Mirick D.K., Chen C., Stanczyk F.Z. (2012). Night shift work and hormone levels in women. Cancer Epidemiol. Biomark. Prev..

[28] Cagnacci A., Paoletti A.M., Soldani R., Orrù M., Maschio E., Melis G.B. (1995). Melatonin enhances the luteinizing hormone and follicle-stimulating hormone responses to gonadotropin-releasing hormone in the follicular, but not in the luteal, menstrual phase. J. Clin. Endocrinol. Metab..

[29] Sato M., Kanikowska D., Iwase S., Shimizu Y., Nishimura N., Inukai Y., Sato M., Sugenoya J. (2013). Seasonal differences in melatonin concentrations and heart rates during sleep in obese subjects in Japan. Int. J. Biometeorol..

[30] Walters J.F., Hampton S.M., Ferns G.A., Skene D.J. (2005). Effect of menopause on melatonin and alertness rhythms investigated in constant routine conditions. Chronobiol. Int..

[31] Nagai M., Tomata Y., Watanabe T., Kakizaki M., Tsuji I. (2013). Association between sleep duration, weight gain, and obesity for long period. Sleep Med..

[32] Knutson K.L. (2010). Sleep duration and cardiometabolic risk: A review of the epidemiologic evidence. Best. Pract. Res. Clin. Endocrinol. Metab..

